# Exploring Retention Strategies for Mid-Career Nurses: A Scoping Review and Thematic Analysis

**DOI:** 10.1177/08445621261463085

**Published:** 2026-06-29

**Authors:** Victoria Babysheva, Elena Neiterman

**Affiliations:** 1 8430 School of Public Health Sciences, University of Waterloo, Waterloo, Canada

**Keywords:** nurses, career stage, health personnel, personnel retention

## Abstract

**Background:**

Healthcare systems face difficulties in retaining nurses due to the demanding nature of the work. Turnover among mid-career nurses is of particular concern, as it diminishes the pool of experienced staff available to provide patient care and mentorship. While retention strategies for early-career nurses are well-documented, less is known about interventions targeting mid-career nurses.

**Methods:**

We conducted a scoping review following Arksey and O’Malley's framework, systematically searching PubMed and CINAHL, including grey literature sources, for studies on mid-career nurse retention published between January 2014 and February 2025. Our goal was to map the scope, nature, and focus of the existing research, and to identify key knowledge gaps.

**Results:**

Twenty-two studies from 10 countries met the inclusion criteria, with 16 examined interventions tailored for mid-career nurses. Among these, ten focused on retaining nurses within their current roles, while six aimed to reduce attrition from the organization entirely. The interventions were grouped into three thematic areas: optimizing role satisfaction, work culture, and customized retention strategies. While these strategies appear promising, their actual impact on retention remains unclear. Many studies provided limited outcome data, lacked consistent measures at both individual and organizational levels, and rarely addressed potential unintended effects. Moreover, there is no widely accepted definition or clear conceptualization of what constitutes a mid-career nurse.

**Conclusions:**

Our findings highlight the lack of focused initiatives to retain mid-career nurses and point to an urgent need for innovation in this space. Future efforts should prioritize the formal evaluation of existing programs to assess their effectiveness.

## Background

Nurses serve at the frontline of healthcare organizations worldwide, delivering patient care as integral members of interprofessional teams (Drennan & Ross, 2019; World Health Organization, n.d.). The nursing workforce comprises professionals who hold different roles, titles and designations across a wide array of healthcare sectors. Nurses are not a homogeneous group. They represent individuals with varying demographic profiles, diverse experiences, and at distinct life-cycle stages (Tourangeau et al., 2014).

The demanding and high-stress nature of the nursing environment presents considerable challenges in both recruiting and retaining nurses (Lavoie-Tremblay et al., 2019; Marć et al., 2019). In 2023, the global nursing deficit was estimated at 5.8 million (World Health Organization, 2025), and shortages are expected to worsen (Drennan & Ross, 2019; Lavoie-Tremblay et al., 2019). Countries within the Organization for Economic Co-operation and Development (OECD), including Australia, Canada, and the United Kingdom, were already experiencing high nurse turnover before the COVID-19 pandemic, with projected shortages estimated at 2.5 million nurses by 2030 (Scheffler & Arnold, 2019).

The COVID-19 pandemic further compounded the healthcare human resources crisis, with many nurses transitioning to other roles, retiring early, or leaving the profession entirely (Canadian Federation of Nurses Unions, 2022; Jackson et al., 2020; Tomblin Murphy et al., 2022). In a 2024 global survey conducted by the International Council of Nurses (ICN), 48.4% of respondents who were members of national nursing associations reported a moderate or significant increase in the number of nurses leaving healthcare (ICN, 2025).

The costs associated with nurse turnover are substantial, both economically and in terms of patient safety (Kelly et al., 2021; Kok et al., 2020; McHugh et al., 2016; Torres et al., 2024), underscoring the urgent need for effective retention strategies. Retention refers to employees remaining in their roles, which may also include registered nurses who change positions within the same organization (Lee et al., 2018). In contrast, turnover involves employees leaving their job or exiting the organization entirely (Hom et al., 2017). In this review, we considered: (1) retention within the same role or unit, (2) retention within the same organization but in a different role, and (3) retention within the nursing profession more broadly. For retention strategies, we considered interventions that establish and maintain a work environment that supports employees staying in their roles by implementing policies and practices that address their needs (Xuecheng et al., 2022).

Experienced mid-career nurses, recognized for their clinical expertise and valuable experience, are often tasked with a disproportionate share of responsibilities, including managing complex patients, supervising students, and supporting peers (Ko et al., 2024; Lobo et al., 2012). The turnover of mid-career nurses exacerbates the shortage of experienced staff at the bedside and results in the loss of institutional knowledge and mentorship for early-career nurses (Hirschkorn et al., 2010; Lartey et al., 2014). Recognizing this, our scoping review focused on mid-career nurses as a cohort warranting targeted retention efforts.

While various person-directed and organizational strategies for nurse retention show promise (Halter et al., 2017; Rickard et al., 2012), their impact and whether these interventions align with their workforce's needs and preferences remain largely unassessed. Many organizations fail to evaluate their effectiveness and outcomes adequately (Twigg & McCullough, 2014), hindering comprehensive understanding and implementation efforts.

Research exists on retention interventions for early-career nurses (Brook et al., 2019; Buckley et al., 2025). However, there is limited research on strategies aimed at retaining experienced mid-career nurses in healthcare settings (Lartey et al., 2014). This scoping review aims to address this gap by exploring existing evidence on retention strategies for mid-career nurses and identifying areas for further research.

The findings of this study have the potential to influence healthcare policies and drive advocacy efforts, emphasizing the importance of nursing retention strategies for enhancing nursing quality of care, ensuring patient safety, and achieving better patient outcomes.

## Methods

The research team utilized Arksey and O'Malley’s (2005) methodological framework to conduct a scoping review of the literature on retention strategies for mid-career nurses. The study aimed to systematically identify the existing body of evidence on the topic, map the breadth and nature of the studies, and determine any gaps in knowledge. Global inclusion was intentional to capture the scope of evidence and identify contextual variation.

### Step 1. Identifying the Research Question

The primary research question was: “What is known about the retention strategies for mid-career nurses?” To ensure a comprehensive study and to gather data that can be used to address the shortage of experienced healthcare professionals, two additional questions were included:

1. What types of retention interventions have been implemented for mid-career nurses worldwide?
2. How effective are these interventions at retaining nurses, and do they have the potential to be replicated?

### Step 2. Identifying Relevant Studies

We gathered worldwide peer-reviewed and grey literature on retention strategies for mid-career nurses published in English from January 2014 to February 2025. Our search was conducted on PubMed and CINAHL. The decision to focus on literature from the most recent decade was due to the significant changes in global health development initiatives, the evolving nature of healthcare delivery with increased digitalization and improved health analytics, changes in nursing practice, and the ongoing shortage of healthcare workers over the last decade. This timeframe also considers the departure of nurses from the profession during the COVID-19 and post-pandemic. The search databases were identified in consultation with a professional librarian, and terms included keywords such as mid-career, experienced or seasoned nurse, retention and strategy (see Table 1). The final search was completed on February 28, 2025, and the review followed the PRISMA reporting standards (Tricco et al., 2018), as outlined in the PRISMA checklist provided in the Supplementary Material (Table S1).

**Table 1.** Terms Used for the Literature Search.

(mid career nurses OR experienced nurses OR mid-career nurses) AND (nursing staff) AND (retention OR turnover OR “intent to stay” OR retain*) AND (in service training OR intervention* OR strategies[title] OR mentorship OR “peer support” OR “leadership development” OR “job embed*” OR Program* OR workshop* OR “work engagement” OR recognition* OR opportunities for development) (mid career OR experienced) AND (nurses OR nursing staff) AND (retention OR turnover OR “intent to stay” OR retain*[ti]) AND (in service training OR intervention*[ti] OR strategies[title] OR mentorship OR “peer support” OR “leadership development” OR “job embed*” OR Program* OR workshop*) (mid career OR experienced OR expert* OR seasoned) AND (nurses OR nursing staff) AND (retention OR turnover OR “intent to stay” OR retain*[ti]) AND (in service training OR intervention*[ti] OR strategies[title] OR mentorship OR “peer support” OR “leadership development” OR “job embed*” OR Program* OR workshop* OR eval*[ti] OR recognition*)

Grey literature search included government and union publications from Canada, the United States, the United Kingdom, Australia, and selected OECD countries represented in the included studies. These searches were conducted through targeted website review and keyword searches rather than a fully systematic grey literature strategy. Dissertations were identified through ProQuest. Relevant papers were imported into Covidence for independent screening and were reviewed by both authors. While this approach enhanced breadth, it does not represent an exhaustive grey literature review.

### Step 3. Selecting Studies to be Included in the Review

This scoping review focused on the population of experienced, expert, seasoned and mid-career nurses who had worked for three or more years and were under the age of 55. This age limit was chosen since age 55 and above is frequently used in literature to describe late-career nurses (Buchan et al., 2020; Canadian Institute for Health Information, 2020; Centers for Disease Control and Prevention, 2012; Stewart et al., 2020). We excluded articles that described nurses as early or late-career and/or did not include information about their job tenure.

Regarding the setting and context, we considered acute care, ambulatory care, healthcare centers, academic, community hospitals and long-term care.

Table 2 provides a detailed outline of the inclusion and exclusion criteria applied in the scoping review.

**Table 2.** Inclusion and Exclusion Criteria.

**Criteria**	**Inclusion**	**Exclusion**
Population	Experiences nurse Expert nurse Mid-career nurse Registered nurse Seasoned nurse	Advanced practical nurse Early-career nurse Late-career nurse Licensed practical nurse (LPN) Midwife New grad Novice nurse Nurse practitioner Nurse in academia Nurse manager Nursing student PhD nurse Paramedic Pre-retirement nurse Registered practical nurse (RPN)
Type of design	Quantitative Qualitative Mixed methods	
Language	English	Articles not in English
Year of publication	January 2014 – February 2025	Studies published before January 2014
Setting/context	Acute care Ambulatory care Community and academic hospitals and healthcare centers Community care Critical care Long term care	Abortion Care Nursing homes Rural and remote
Intervention	Any additional training to improve or advance skills, including retraining, simulation training, training for another nursing specialty, etc. Assignments for non-clinical work, non-patients care roles and/or activities Extra staffing Flexible scheduling Huddles Introduction of support teams (e.g., interdisciplinary, new models of care, etc.) to augment nursing Leadership development Mental health support Mentorship Opportunities for career development and/or advancement Peer support Preceptorship Recognition Retention strategies Strategies to reduce physical toll (e.g., by adding nurse extenders, equipment support, etc.) Wellness activities Workshops	Compensation
Outcomes	Improved retention Improved retention measures Improved intent to stay Reduced turnover	Attrition Recruitment
Study characteristics	Systematic reviews Scoping reviews Rapid reviews Narrative reviews Case studies Reports (e.g., industry and/or government reports, nurses’ unions or regulatory bodies reports, etc.) Critically appraised topics (e.g., evidence synthesis, guidelines, evaluations, assessments of interventions, etc.) RCTs Cohort studies Case-Control studies Case series	Letters to editors Guidelines Study protocols Expert opinions

In total, 892 references were imported into Covidence, and 255 were identified as duplicates and removed. Additionally, 18 studies (2%) were added through citation search by screening through the reference lists of included studies.

Initially, 637 titles with corresponding abstracts were screened, leaving 61 studies (10%) for full-text review. The researchers (VB and EN) met several times to clarify inclusion and exclusion criteria and discuss points of agreement and disagreement during the title and abstract screening process.

Out of the 61 articles, 39 (64%) were found to be outside the scope of this review due to participant population, study settings, context, lack of description of strategies, outcomes, or interventions. Articles that described retention strategies for early- or late-career nurses underwent full-text review to ensure they did not include nurses who met our inclusion criteria. The final screening resulted in 22 articles included in the present review. The results from the search and selection of articles are presented in the PRISMA flow diagram (see Figure 1).

**Figure 1.**
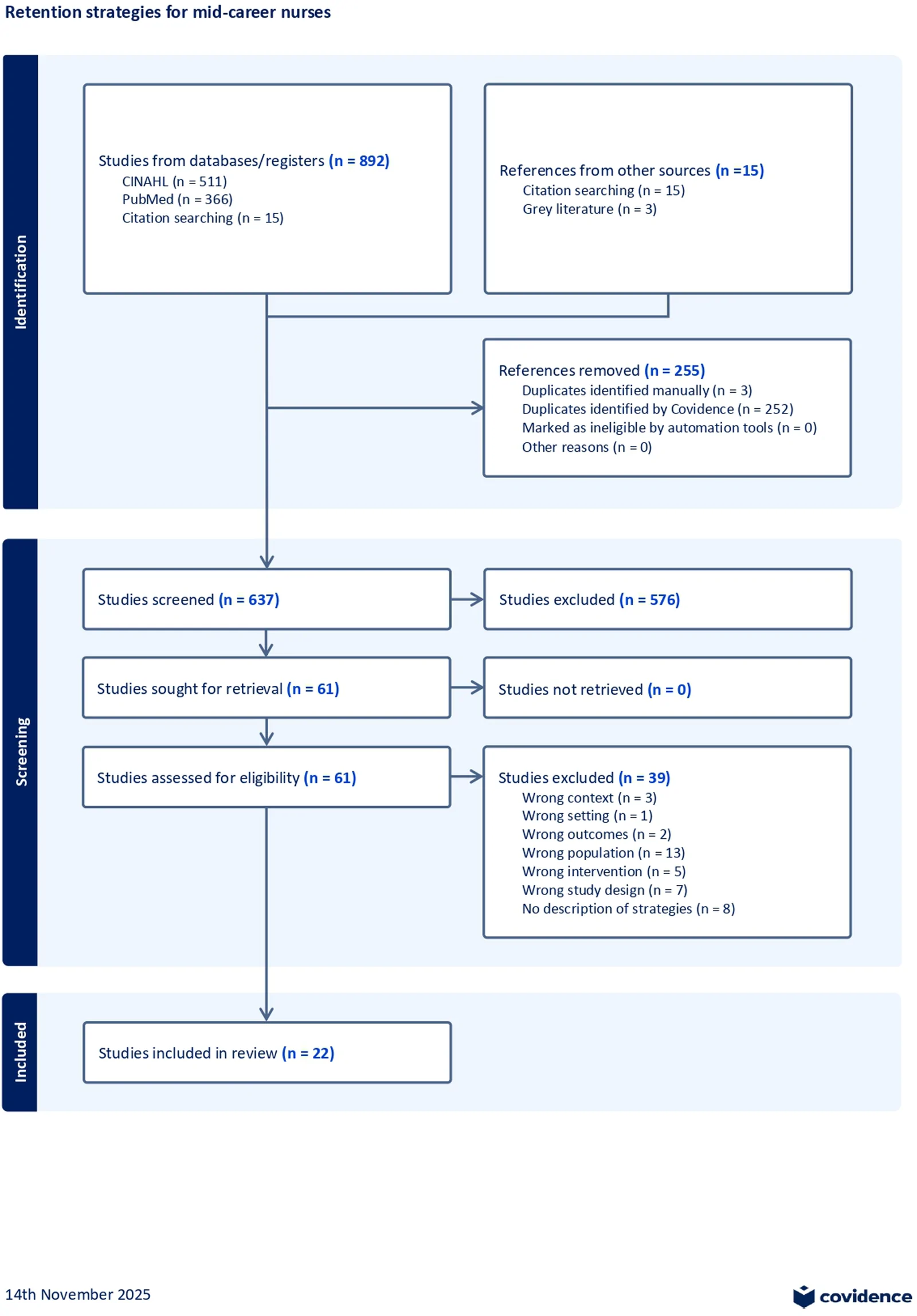
PRISMA Flow Diagram.

### Step 4. Charting Data

The Microsoft Excel data extraction tool was developed and included the following categories: author, year, country, study aims and design, sample size and characteristics, context, intervention, and main findings. Once data was extracted from 10 papers, the researchers (VB and EN) met to review the tool and refine the form.

### Step 5. Collating, Summarizing, and Reporting Findings

After gathering data from selected articles, we conducted a thematic analysis (Braun & Clarke, 2006). This involved analyzing excerpts of the articles in relation to research objectives to identify key themes. The authors practiced reflexivity, engaged in consensus discussions, and conducted auditing to ensure rigor. Each study was inductively coded without an a priori framework, to avoid imposing pre-established assumptions on the literature. Through this process, the authors discussed the findings and agreed on the final coding scheme, resulting in the development of the following themes: optimizing role satisfaction, work culture and customized retention strategies.

## Results and Findings

Twenty-two studies from 10 countries were included in the scoping review: Canada: n = 2, UK: n = 3, USA: n = 10, China: n = 1, Indonesia: n = 1, Netherlands: n = 1, Sweden: n = 1, Korea: n = 1, Denmark: n = 1 and Oman: n = 1. Sample sizes ranged from 9 to 1146.

Care settings varied in adult care and included a single study for nurses working with the pediatric population (Gilroy et al., 2020). Only one study was specific to male nurses (Shin & Lim, 2021), while others had male and female participants.

Retention strategies for mid-career nurses were examined directly or indirectly in all studies. The latter refers to interventions aimed at improving factors such as job satisfaction, empowerment, or professional development, where retention was measured as a secondary outcome rather than the primary endpoint. The main characteristics of the included studies are represented in Figure 2.

**Figure 2.**
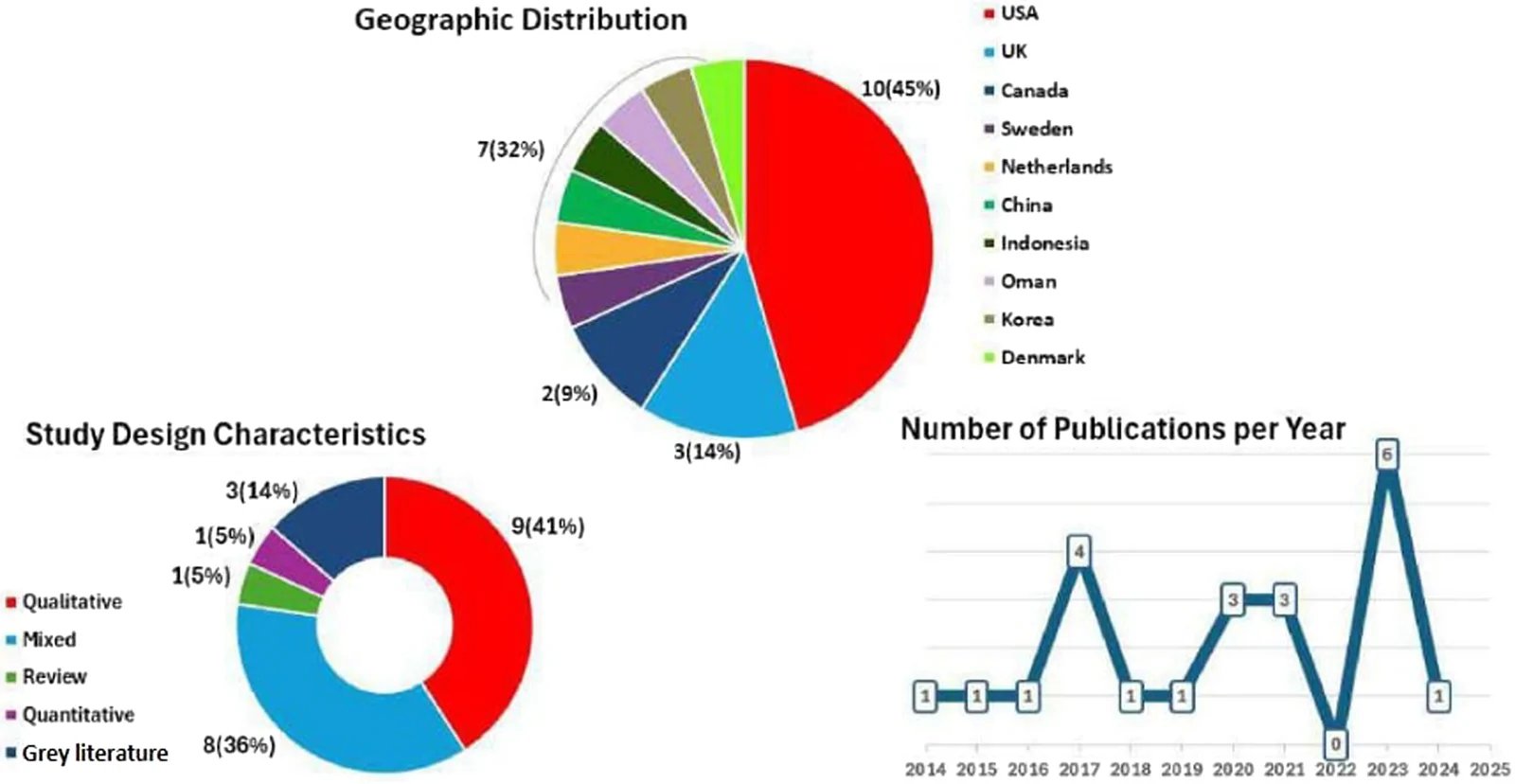
Characteristics of Included Studies.

In terms of methodology, there were qualitative (n = 9), mixed (n = 8), quantitative (n = 1) studies and a rapid review (n = 1). The main findings for each study are summarized in Table 3.

**Table 3.** Summary of the Included Papers.

Authors, Year	Country	Study Aim(s)	Study Design	Sample Size & Key Characteristics	Context	Intervention	Main Findings
Allen, 2017	USA	Develop solutions that will negate the adverse effects of contributing factors in the turnover rate of experienced nurses in the inpatient hospital setting	Mixed (surveys)	Experienced nurses (n = 10)	Hospital	Evidence-Based Mentorship Program for Experienced Nurses	An experienced nurse mentorship program was implemented to support nurse retention, emphasizing strong professional relationships and organizational commitment. The structured, year-long program is tailored to the organization's needs and relies on the continued involvement of nurse champions to sustain its impact. Its goals included reducing turnover, improving patient outcomes, and lowering organizational costs.
Bélanger-Hardy et al., 2024	Canada	1. Review evidence on how nurse mentorship programs impact turnover and retention across early-, mid-, and late-career nurses in acute care. 2. Identify features that contribute to program success or failure	Review (rapid)	860 articles	Hospital, inpatients & acute settings	Mentorship programs	The existing literature lacks reviews focused on mentorship programs for mid-career nurses, who are often grouped with late-career peers despite having distinct needs. Though experienced and capable of long-term contributions, mid-career nurses receive limited attention and report lower job satisfaction than early- and late-career colleagues.
Dawood & Gamston, 2019	UK	Explore why nurses were turning away from the Emergency Departments (ED) and what alternatives they considered	Qualitative (surveys)	Nurses (n = 12), males(n = 5) and females (n = 7), years of experience - from 3 to more than 10	Emergency departments	Work- based program designed and delivered in collaboration with a local health education institute	Engagement plays an important role in retaining ED nurses. It is shaped by practice environment, staffing, and relationships. Stress from care quality and patient flow issues highlights the need for alternative roles. Offering full or part-time ENP positions can help retain nurses at risk of leaving. Sustainable strategies, including dual roles and incorporating nurses’ input, are key to making ED nursing a viable long-term career.
Embree et al., 2015	USA	1. Describe an example of a nursing sabbatical. 2. Postulate the merits of a balanced scorecard (BSC) with strategy map (SM) in measuring the nursing sabbatical	Mixed (pre- and post- assessment measures such as nurse vacancy and turnover rates, RN satisfaction, patient experience surveys, balanced scorecard with strategy map)	Not described	Acute care	A nursing sabbatical program and an innovative framework for measuring sabbatical programs	Sabbaticals are proposed as an innovative approach to attract and retain skilled RNs, offering opportunities for professional growth and renewal. Implementing a nursing sabbatical program in acute healthcare organizations has the potential to enhance employee experience, combat burnout, retain talented nurses, and improve quality of care, productivity, and commitment.
Evans, 2017	UK	Address a critically high nursing vacancy rate in ED	Mixed	Not described	Emergency Department	A structured recruitment and retention strategy included educational initiatives for both junior and experienced nurses, a preceptorship program for new recruits, clearer leadership and governance structures, and the addition of associate and trauma practitioner roles.	Innovative retention strategies included introducing specialist higher education courses and new roles such as associate practitioners and trauma practitioners. Collaboration with local universities and educational initiatives aimed at challenging misconceptions about working in the emergency department were also key components. The outcomes of these efforts were promising, with a significant reduction in the vacancy rate and successful recruitment of new nursing staff. The success of the initiative highlights the importance of proactive strategies tailored to the specific needs of healthcare areas, suggesting the potential for similar approaches to be effective in other contexts.
Folden et al., 2023	Denmark	Explore nurses’ perception of nurse turnover and retention	Qualitative study (table-top simulations)	15 workshops involving a total of nurses (n = 43), including experienced nurses (n = 13)	Internal medicine departments	Table-top simulations provided data on the nurseś perspectives of turnover and retention	Experienced nurses highlighted the need for collaborative work environments characterized by strong collegial relationships with peers, consistent support and constructive feedback from supervisors. They also highly value lifelong learning opportunities, focusing on quality of care, task variations, and exposure to clinical challenges are all important for this group of nurses. Empowering experienced nurses to influence their work-related tasks and involving them in decision-making processes further contributes to enhanced job satisfaction and retention rates.
Gilroy et al., 2020	USA	Prevent outward migration of experienced nurses	Mixed (surveys)	Pediatric nurses (n = 35) with average length of experience 3.7 years (median = 3 years, range: 2–9 years)	Children's hospital	The Bridge Program was implemented to increase retention by facilitating career development and specialization in pediatric critical care	The desire to transition to a higher level of care, such as the ICU, was identified among experienced nurses. The program's success was attributed to its nurse-driven approach, which fostered informal learning and relationship-building. Cohort-based learning facilitated collaboration and communication among participants. The program catered to nurses at a critical turnover risk, providing support beyond the initial year of practice. Transparency in program eligibility and progression fostered trust in leadership and managed expectations. The program's structure could serve as a model for transitioning to other specialties or critical care settings, with adjustments tailored to specific needs.
Guo et al., 2020	China	To explore the effects of WeChat- based ‘three good things’ (3GT) on burnout nurses’ turnover intention and coping styles	Quantitative (randomized controlled trial)	Nurses (n = 73), including intervention group (n = 33), males (n = 0) and females (n = 33), and the control group (n = 40), males (n = 1) and females (n = 39). Length of service (Mean ±SD) for intervention group (6.21 ±1.11 years) and for control group (7.63 ±2.84 years)	Tertiary hospital	WeChat-based ‘three good things’ (3GT) intervention focuses on promoting people's well-being and alleviating mental distress by using intentional activities to cultivate their positive cognition and emotions	The study found that practicing WeChat-based 3GT daily was feasible, acceptable, and significantly reduced turnover intention among nurses experiencing burnout. The research identified three main strengths: the minimal time and effort required for 3GT, its implementation through WeChat, which is convenient and enjoyable for nurses, and its effectiveness in promoting positive coping styles. This study enhances our understanding of burnout management and offers a practical approach for nurses and managers to address turnover and burnout issues.
Hayward et al., 2016	Canada	Explore the personal and environmental factors that influenced experienced nurses’ decisions to leave and seek an alternate position in nursing	Qualitative (interviews)	Nurses (12), males (n = 0) and females (n = 12), with average length of experience 16 years (range 5–26 years) who have left nursing positions in acute care	Acute care inpatient settings	No specific interventions	The study explored the reasons experienced nurses decide to leave their positions in acute care. It identified three main factors influencing their decisions: challenging workplace environments, limited leadership support, and personal health issues. Workplace environments, characterized by high patient acuity and increased workload demands, contribute significantly to nurses’ stress and burnout. Professional relationships play a crucial role, with ineffective communication and lack of support exacerbating feelings of dissatisfaction. Limited leadership leads to decreased morale and confidence among nurses. Personal health issues resulting from workplace stress also contribute to nurses’ decisions to leave. The findings emphasize the importance of addressing the workplace environment, leadership practices, and nurses’ well-being to retain experienced nursing staff.
Hörberg et al., 2023	Sweden	Explore factors that motivate experienced nurses in the workplace and the underlying reasons for strong intentions to leave and high staff turnover	Qualitative (surveys)	Nurses (n = 1,146), males (n = 111) and females (n = 1,035), between 11 and 15 years since the graduation	Various sectors of the healthcare system	No specific interventions	The study investigated the factors that influence motivation, intention to leave (ITL), and turnover among experienced nurses. It identified five broad categories: organizational characteristics, work characteristics, relationships at work, recognition, and health issues. Motivational factors included clearly defined roles, opportunities for professional influence, and supportive management. Reasons for ITL and turnover encompassed issues such as long working hours, difficulty balancing work and personal life, inadequate staffing, and lack of influence over work. Poor relationships with colleagues, lack of recognition, and concerns about health, including burnout and stress, were significant contributors to ITL and turnover. The study emphasizes the importance of addressing these factors comprehensively to improve motivation, retention, and overall well-being in the nursing profession.
Knighten & Yvanovich, 2023	USA	Suggest a solution to RN outward migration	Grey literature	Not described	Acute care, operating room/ perioperative services	A structured internal model for experienced nurses to transition from hospital patient care units to practice in the specialty of perioperative nursing	Experienced nurses have unique skills but need training in perioperative-specific tasks. Transition to practice programs should include customized educational components, mentorship, and exposure to different perioperative roles. Successful initiatives have shown the effectiveness of academic-practice partnerships in preparing nurses for perioperative practice, ultimately contributing to workforce stability and the strength of the nursing profession. By facilitating transitions, healthcare organizations can retain valuable talent and improve patient care outcomes.
Kramer et al., 2021	USA	1. Understand the nurse mentoring culture within a hospital environment. 2.Develop a mentoring model that will guide and support a mentoring program	Qualitative (semi- structured focus groups interviews)	21 focus groups included nurses (n = 42), males (n = 4) and females (n = 38), with a mean of 22 years of experience (SD= 11.99, range = 4–50)	Acute care hospital settings	The Art of Nurse Mentoring Model is a supportive mentoring framework that was developed from the data	The study highlights the significance of evidence-based outcomes of mentoring and its impact on individual nurses, unit culture, and organizational effectiveness. Clear guidelines, education, and role clarification are important for establishing a culture of mentoring. Mentoring is viewed as integral for the personal and professional growth of nurses, offering potential benefits such as increased trust, confidence, and collaboration, which can lead to improved retention rates.
Kuykendall et al., 2014	USA	1.Examine the level of engagement in direct care nurses 45 years or older with at least 10 years’ experience 2.Compare engagement levels of nurses participated in Fanning the Flame (FTF) program with those who had not.	Mixed (surveys)	Direct care nurses (n = 142) 45 years or older with 10 or more years’ experience	Tertiary care medical center	Fanning the Flame program was internally developed to reward, renew, and revitalize experienced nurses	Experienced nurses play a critical role in promoting positive patient outcomes and mentoring novice nurses. Creating an engaged nursing workforce involves monitoring work engagement levels among nurses. This information should be used to establish multiple strategies and programs to retain, re-energize, and engage experienced nurses. Engaged experienced nurses are more likely to pursue advanced degrees and certifications, have discussions about their career paths, and experience professional growth. Utilizing existing resources such as unit and organizational shared decision-making councils provides a platform for nurses to contribute to organization-specific solutions and offer feedback and suggestions for improving the work environment
Maqbali, 2018	Oman	1.Explore critical care stakeholders’ suggestions to improve the retention of intensive care unit (ICU) nurses. 2.Compare and contrast suggestions by role. 3.Analyze how the findings differed from the value profile results reported in the literature	Qualitative (semi- structured interviews)	21 healthcare professionals: nurses (n = 7), physicians (n = 7), healthcare administrators (n = 7)	Intensive care units	The research assessed the potential use of value profiles as a tool for human resource management to improve the retention of critical care nurses	It was found that nurses have changing needs and priorities over the course of their careers, which highlights the importance of implementing customized retention strategies. The study suggests that targeted approaches tailored to specific nurse profiles are more effective than generic solutions. In addition, the author recommends involving frontline nurses in decision-making processes to develop strategies that effectively address their concerns.
National Health Service Employers, 2024	UK	1.Improve retention at all stages of nurses’ careers. 2.Reduce nursing turnover at the trust by one per cent over a 12-month period.	Grey literature	Not described	Hospitals	A retention framework based on identified groups within the workforce	The recommendation is to customize retention initiatives based on staff needs and motivations. It is also suggested to set up a dedicated retention project team to create an action plan with clear objectives, roles, and responsibilities. Engaging with staff for input and feedback on implemented changes is emphasized.
Rogers & Burke, 2021	USA	Redesign and evaluate the orientation program for experienced registered nurses, creating a learner- centered environment to enhance engagement, satisfaction, and retention.	Mixed (surveys)	Experienced nurses (n = 161)	Hospitals	The Experienced RN Orientation Program (ERNOP) was redesigned to increase participant engagement by incorporating multiple learning modalities, with consideration for individual learning Styles.	The revised orientation program emphasizes engagement and interactivity and is tailored to meet experienced nurses’ learning needs. Incorporating various teaching methods and ensuring facilitators possess essential facilitation skills and a positive attitude contribute to program success. Continuous evaluation, innovation and adaptation based on participant feedback are crucial for designing effective nursing orientation programs that support retention and satisfaction. Ongoing studies on performance and retention show promising early results.
Seller-Boersma et al., 2023	Netherlands	1.Explore how hospital nurses perceive working conditions that affect their retention 2.Enhance knowledge of relevant elements of retention 3.Aid in the development of retention interventions in hospital settings	Qualitative (semi- structured focus group interviews)	13 focus groups’ interviews with total participants (n = 93), including 2 focus groups with experienced nurses (n = 10)	Academic hospitals, teaching hospitals, employment agency, Universities of Applied Sciences, and a foundation that focuses on increasing the labor potential of nurses	No specific interventions	The study identified three main themes that influence how nurses perceive their working conditions in hospitals in relation to retention: finding a personal pathway, constructive collaboration, and the availability of supporting facilities. It emphasized the importance of understanding factors at individual, team, and organizational levels to develop effective retention strategies. The study also highlighted the significance of nurses’ autonomy and control, as well as the role of managers in nurse retention. Structural organizational empowerment and supportive management are crucial factors contributing to nurse retention. Nurses emphasized the multifactorial nature of retention factors, suggesting that interventions targeting specific elements can have consequential effects on other aspects.
Shin & Lim, 2021	Korea	1.Clarify the lived experiences of mid-career male nurses in clinical practice; 2.Provide data to help devise a working environment and organizational culture that fosters male clinical nurses’ long-term job retention	Qualitative (semi- structured interviews)	Male registered nurses (n = 9) with a minimum of five years’ work experience in a general hospital	Emergency rooms, operating rooms, a psychiatric ward, an internal medicine ward, and an outpatient cardiac center	No specific interventions	The study examines the challenges that male nurses face when adapting to the hierarchical clinical culture, which is predominantly female-dominated. Male nurses have expressed concerns about limited opportunities for career advancement and uncertainty about their future in the profession. They also struggle with confidence issues regarding their competence and feel pressure to actively demonstrate their expertise. The findings suggest that transitioning to a more cooperative and inclusive organizational culture would improve the working environment for male nurses. This underscores the need for nursing administrators to develop effective policies to retain and support male nurses and to provide them with opportunities in clinical settings.
Shinners, 2017	USA	Examine the use of a professional development assessment tool to facilitate discussions for career pathway support and retention of experienced RNs	Grey literature	Not described	Healthcare organizations (Not specified)	The RN Professional Development Assessment (PDA) tool was created to offer RNs the chance to explore potential professional development across four career paths within the organization: administration, clinical expert, nursing education, and research.	The development of a PDA facilitates discussions between the organization and registered nurses who are seeking career advancement, helping them become aware of available roles and positions. Implementing an organization-specific PDA could serve as a strategy to retain experienced staff who are looking to expand their current role. When consistently used as a basis for professional development discussions, the PDA can reduce the desire of experienced RNs to leave by providing internal opportunities.
Torres et al., 2024	USA	Improve RN organizational retention through professional development opportunities	Mixed (evaluation)	Nurses (n = 30), males (n = 2) and females (n = 28), with a minimum of 2 years of experience at the organization	The program was implemented at a 938-bed quaternary medical center in the Midwest region of a large nonprofit healthcare system in the United States	Best Fit for Nurse Advancement (BFNA) is a program that provides dedicated time, resources, and support for experienced nurses to explore career advancement pathways.	The BFNA program has been effective in promoting career advancement and retaining experienced registered nurses. It offers dedicated time and resources for nurses to explore professional development opportunities and reflect on their individual strengths. Program outcomes have included increased organizational retention, career growth, and a positive return on investment.
Wardhani & Hariyati, 2023	Indonesia	Explore the current experience of Indonesian nurses who retain their jobs in the hospital setting	Qualitative (semi- structured interviews)	Nurses with more than five years of experience (n = 10), males (n = 3) and females (n = 7)	Hospital	No specific interventions	Maintaining positive relationships with colleagues, effective communication, and providing rewards and recognition are essential for retaining experienced nurses. Offering professional development opportunities, leadership support, adequate compensation, workload management and clear career paths can also motivate nurses to stay.
Yarbrough et al., 2017	USA	Explore relationships of professional values orientation, career development, job satisfaction, and intent to stay in recently hired mid- career and early- career nurses in a large hospital system	Mixed (surveys, interviews)	Nurses (n-67), including mid- career nurses (n = 9)	Hospital	No specific interventions	The study found that newly hired mid-career nurses are more likely to continue employment compared to early-career nurses. It also noted intergenerational tensions in perceptions of dedication to the profession and workplace commitment. The study recommends intergenerational collaboration to address retention concerns and highlights the need for tailored retention strategies to meet the diverse needs of nurses across career stages.

The majority of studies, 16 (73%), described specific retention intervention projects for mid-career nurses. Of these studies, the types of the interventions could be divided into improving retention for the same position (n = 10) or preventing outward migration of nurses from the organization (n = 6).

The first category of the interventions with the goal of retaining nurses to work in the same unit and role included three mentorship programs (Allen, 2017; Bélanger-Hardy et al., 2024; Kramer et al., 2021), three multifaceted customized strategies addressing the needs of specific sub-groups of mid-careers nurses (Folden et al., 2023; Maqbali, 2018; United Lincolnshire Hospitals NHS Trust, 2020), one reward initiative (Kuykendall et al., 2014), one orientation project (Rogers & Burke, 2021), one wellness program (Guo et al., 2020) and one project targeting professional development for the same unit and job role (Embree et al., 2015).

The purpose of the second category of retention initiatives was to prevent outward migration of human resources outside the organization, and it was represented by four programs for nurses considering switching to alternate nursing roles (Dawood & Gamston, 2019; Evans, 2017; Shinners, 2017; Torres et al., 2024) and two transition programs to continue working in the nursing role at another level of care or unit in the same healthcare organization (Gilroy et al., 2020; Knighten & Yvanovich, 2023).

Within each of the three main themes (optimizing role satisfaction, work culture and customized retention strategies) there were also identified subthemes, summarized in Figure 3.

**Figure 3.**
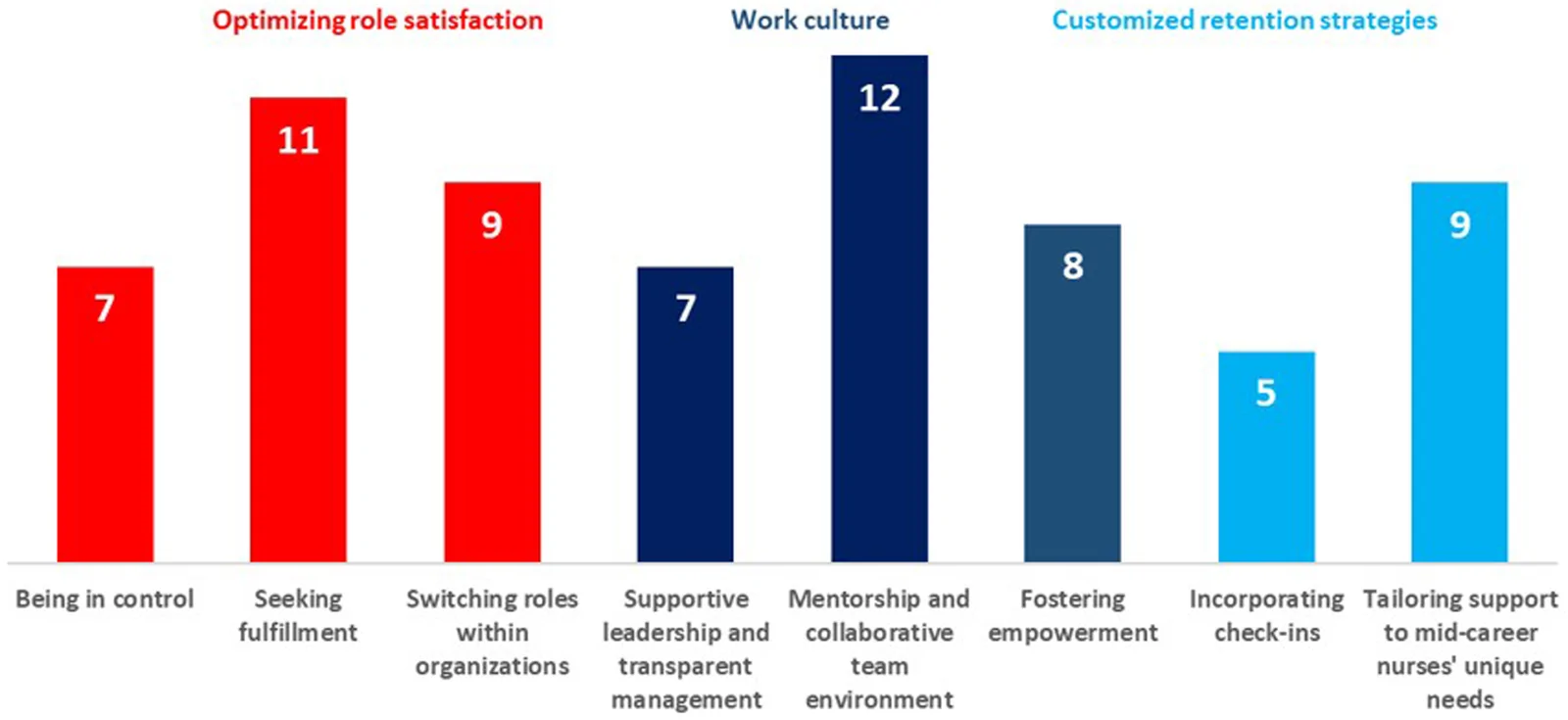
Summary of the Prevalence of Sub-Themes within Each Thematic Group.

### Theme 1. Optimizing Role Satisfaction

The overarching theme of optimizing role satisfaction describes the core elements influencing nurses’ fulfillment of professional roles and aspirations and the desire to remain in their positions. It emphasizes the significance of autonomy, career advancement opportunities, and diverse career pathways in improving job satisfaction and retention among mid-career nurses within healthcare organizations. In this thematic category, we coded 16 articles that included the subthemes of “Being in control” over nursing work (n = 7), “Seeking fulfillment” of desire for career progression (n = 11), and “Switching roles within organizations” to alternative nursing positions (n = 9).

#### Being in Control

This subtheme underscores the role of autonomy and control over professional practice in retaining mid-career nurses (Hayward et al., 2016; Hörberg et al., 2023; Seller-Boersma et al., 2023; Wardhani & Hariyati, 2023). A study of nurses’ perception of turnover and retention in Denmark demonstrated that experienced nurses who feel that they have influence over their daily tasks are less likely to leave their roles (Folden et al., 2023). It showed that nurses prioritized the quality of patient care and valued clinical challenges, seeking positions with flexible planning and input into the organization of their work.

Factors such as autonomous decision-making, alignment with personal values, and the ability to influence work routines and schedules were identified as motivational (Hörberg et al., 2023). Giving control over nursing work and offering professional development opportunities were also recognized by the article from the Netherlands as non-monetary incentives that enhanced job satisfaction (Seller-Boersma et al., 2023). Experienced nurses, as highlighted by studies in this sub-theme, wanted to feel valued and have ownership over their practice (Allen, 2017; Torres et al., 2024).

#### Seeking Fulfillment

Studies in this subtheme underlined the importance of career pathways and opportunities for career progression that align with mid-career nurses’ aspirations, with “seeking fulfillment” meaning achieving personal and professional satisfaction as a nurse (Folden et al., 2023; Hörberg et al., 2023; Seller-Boersma et al., 2023; Wardhani & Hariyati, 2023). One article demonstrated a correlation between career fulfillment, a positive workplace atmosphere, and nurse retention, indicating the significance of career advancement opportunities (Gilroy et al., 2020).

A study of critical care nurses in Oman concluded that as nurses gain experience, their interest in professional development and education increases (Maqbali, 2018). Factors that motivate career progression among mid-career nurses included satisfaction from clinical roles, skill development, and the transferability of acquired skills (Dawood & Gamston, 2019). Personalized career advancement plans were found to enhance communication between organizations and experienced nurses, leading to better awareness of available roles and positions (Shinners, 2017). Engaged nurses were more likely to pursue advanced degrees, discuss potential career paths, and experience professional growth, ultimately benefiting patient care (Kuykendall et al., 2014).

Safe learning environments and clear career paths favourably influenced job satisfaction and experienced nurses’ retention (Seller-Boersma et al., 2023; Torres et al., 2024; Wardhani & Hariyati, 2023). Challenges such as perceived job growth limitations, particularly felt by male nurses, spotlighted the need to promote diversity in leadership roles and to continue supporting career aspirations (Shin & Lim, 2021).

#### Switching Roles Within Organizations

Articles under this subtheme shed light on the trend of nurses considering diversifying or expanding their current roles or pursuing alternative positions within healthcare organizations. The desire of mid-career nurses to transition to alternate nursing roles or different units, including specialty or intensive care units, suggested a need for diverse nursing employment opportunities (Evans, 2017; Gilroy et al., 2020). It was noted that nurses might consider changing job roles as part of preparing for a better future, indicating a proactive approach to career development (Shin & Lim, 2021).

A study from the UK found that retaining experienced nurses in high-stress areas like emergency care (Evans, 2017) could be a challenge. However, this could be overcome by offering dual roles, such as combining emergency department (ED) nursing with an emergency nurse practitioner role, to retain nurses who find the constant pressures of the ED exhausting (Dawood & Gamston, 2019). One article stressed the importance of clinical nurse specialists actively participating in clinical work, not just managerial duties, to maintain the quality of patient care (Folden et al., 2023).

Three articles from the US highlighted the need for organizations to develop plans to advance nurses into new roles in order to retain them (Knighten & Yvanovich, 2023; Shinners, 2017; Torres et al., 2024). For example, one study found that professional development programs for experienced nurses foster organizational loyalty by offering pathways to roles beyond direct clinical care (Torres et al., 2024). Knighten and Yvanovich (2023) proposed the creation of an internal workforce pipeline to stem the outward migration of nurses, promoting resilience and facilitating transitions to perioperative specialty practice. Several healthcare facilities in the UK implemented initiatives such as showcasing career pathways through video case studies and introducing an internal transfer policy to encourage staff to move to different roles within the hospital, aiming to retain experienced nursing staff within the organization (United Lincolnshire Hospitals NHS Trust, 2020).

### Theme 2. Work Culture

The work culture theme encompasses the organizational atmosphere and interpersonal dynamics that shape nurses’ experiences and influence their decision to stay in their roles. It was discussed in fifteen articles and was coded with the following subthemes: “Supportive leadership and transparent management” (n = 7), “Mentorship and collaborative team environment” (n = 12) and “Fostering empowerment” (n = 8).

#### Supportive Leadership and Transparent Management

A Dutch study found that support of leadership was paramount for experienced nurses’ retention (Seller-Boersma et al., 2023). Researchers exploring Indonesian nurses’ experiences found that effective leadership styles were characterized by supervisors’ reliability and ability to manage and motivate staff, which positively impacted the work environment and team cohesion, influencing nurse retention (Wardhani & Hariyati, 2023). Supportive leadership behaviour included guidance for autonomous decision-making, acknowledgement and recognition of contributions, transparent communication, and encouragement of professional development (Hayward et al., 2016). A qualitative study of experienced nurses in Canada concluded that challenges in communication and teamwork resulting from inadequate leadership can lead to nurses’ decisions to leave (Hayward et al., 2016).

One study found that nursing expertise was retained when their managers listened to their perspectives (Dawood & Gamston, 2019) as the nurses valued transparent leadership and trust, particularly through programs offering clear career progression paths open to all nurses (Gilroy et al., 2020).

One article from the UK advocated for establishing multidisciplinary learning and development boards to oversee design and implementation of educational initiatives, thereby nurturing workforce development in collaboration with local stakeholders (Evans, 2017). Meanwhile, another UK article suggested actively engaging with staff to solicit input on implemented strategies and providing feedback on successes (United Lincolnshire Hospitals NHS Trust, 2020).

#### Mentorship and Collaborative Team Environment

More than half of the included articles elaborated on how a positive work environment, camaraderie, effective communication, and constructive collaboration at the team level, along with supportive management, contributed to retaining experienced nurses (Dawood & Gamston, 2019; Gilroy et al., 2020; Hayward et al., 2016; Hörberg et al., 2023; Seller-Boersma et al., 2023; Wardhani & Hariyati, 2023; Yarbrough et al., 2017). Several authors emphasized communication as the cornerstone of good teamwork and stressed the importance of a conducive and stimulating learning culture (Folden et al., 2023; Wardhani & Hariyati, 2023).

Passionate and engaged mid-career nurses may be key for mentoring and contributing to organizational success. A US study that examined the level of engagement of experienced nurses identified the need to cultivate collegial support between experienced and novice nurses to enhance retention of both nursing cohorts (Kuykendall et al., 2014).

Mentorship was demonstrated to directly reduce turnover rates among nursing staff while enhancing their proficiency, confidence, and abilities, as well as promoting a culture of support and safety within healthcare environments (Kramer et al., 2021). Mentorship was described as a means of raising professional standards and fostering a sense of belonging among mid-career nurses (Allen, 2017). It was noted that collegiality within engaged and competent teams could keep experienced nurses in their positions longer despite increased workloads and high patient acuity (Hayward et al., 2016).

A study from Korea that focused exclusively on male nurses found that they felt a particular need for support and encouragement from male colleagues or men in more senior nursing roles, as it was difficult for them to form personal relationships with female counterparts (Shin & Lim, 2021).

#### Fostering Empowerment

The retention of mid-career nurses is influenced by the concepts of empowerment and a sense of belonging in the work environment, where nurses feel resilient, optimistic, and fully engaged (Allen, 2017). Organizational empowerment involves providing equal access to information, support, resources, and learning opportunities (Laschinger et al., 2001; Seller-Boersma et al., 2023). A Swedish study concluded that nurse empowerment, whether at an individual or team level, served as a motivational factor that aided in achieving operational goals and delivering high-quality of care (Hörberg et al., 2023).

Leaders who tailored specific strategies to meet the unique needs of mid-career nurses were found to prolong these nurses’ tenure within the organization (Evans, 2017). However, top-down decisions without frontline staff input may lead to strategies failing to address nurses’ concerns (Maqbali, 2018). A UK article suggested that engaging with staff to gather their input and provide updates on successes would be effective for fostering a sense of involvement among mid-career nurses (United Lincolnshire Hospitals NHS Trust, 2020). A study from Denmark found that high-performing hospitals were characterized by flat organizational structures, staff involvement in development policies promoting lifelong learning and career opportunities, and structured educational programs (Folden et al., 2023). The US study concluded that fully engaged nurses significantly contributed to organizational success and proposed that nurse executives should prioritize creating environments where nurses take pride in their work (Kuykendall et al., 2014).

### Theme 3. Customized Retention Strategies

Thirteen articles coded in this thematic category focused on the multifaceted nature of nurse retention and emphasized the importance of analyzing nurses’ profiles to understand their life cycle and career stage needs in order to design and implement customized programs with well-defined objectives and pathways. This approach was described as ensuring that the interventions cater to the unique requirements of nurses at different points of their lives and careers. Two subthemes were included in this thematic category: “Incorporating Check-ins” (n = 5) and “Tailoring Support to Mid-Career Nurses’ Unique Needs” (n = 9).

#### Incorporating Check-ins

Checking-in refers to the initial step in the process where organizations seek to understand the diverse needs of mid-career nurses before crafting and implementing interventions. For instance, critical care nurses have specific workload concerns based on their experience levels as they are assigned to care for patients with more comprehensive needs and often have to assist their less experienced novice colleagues, indicating a need for tailored approaches (Maqbali, 2018). To address these challenges, one of the articles from the UK emphasized the importance of understanding staff preferences to implement initiatives accordingly (United Lincolnshire Hospitals NHS Trust, 2020). Another example from the US was to provide customized training opportunities to retain experienced nurses transitioning to specialized roles within the same healthcare facility, which could help prevent outward migration (Knighten & Yvanovich, 2023).

There are complex relationships between the quality of leadership, nurses’ well-being, and turnover (Hayward et al., 2016). The US study that examined relationships of professional values orientation, career development, job satisfaction, and intent to stay indicated that managers should strive to understand mid-career nurse profiles (e.g., younger experienced nurses versus mature expert nurses) to facilitate targeted interventions and maintain staff work engagement (Yarbrough et al., 2017).

#### Tailoring Support to Mid-Career Nurses’ Unique Needs

The unique needs of mid-career nurses could be met through a combination of strategies, including lifelong learning, fostered collaboration with colleagues and physicians, and active involvement in shaping organizational interventions (Folden et al., 2023; Rogers & Burke, 2021). An article from the UK proposed to simultaneously utilize short-term solutions and long-term investments to improve nurse retention in challenging circumstances (Evans, 2017).

Since there is no one-size-fits-all approach, segmented retention strategies were suggested to address the varying needs of nurses throughout their life-cycle and career stages (Maqbali, 2018). One article described the effectiveness of programs led by bedside nurses (Gilroy et al., 2020), while others advocated for thinking “outside the box” in creating strategies to enhance nurse engagement (Rogers & Burke, 2021) or advised on the use of best practices for organizational-level interventions (Kuykendall et al., 2014). In addition, a Dutch study acknowledged the interconnectedness of factors influencing retention and advocated for interventions targeting multiple elements at individual, team, and organizational levels (Seller-Boersma et al., 2023).

## Discussion

Our scoping review on global retention strategies for mid-career nurses aimed to address a gap in evidence by systematically exploring existing literature on retention strategies for this nursing cohort, mapping the extent and nature of studies, and identifying areas for further research in this critical area of healthcare workforce management.

Three primary thematic categories were identified in the literature: optimizing role satisfaction, work culture, and customized retention strategies. The theme of optimizing role satisfaction highlights the fundamental factors that contribute to nurses’ sense of fulfillment in their professional roles, emphasizing autonomy, career advancement opportunities, and diverse career pathways as crucial elements in enhancing job satisfaction and retention among mid-career nurses. The work culture theme spotlights the organizational atmosphere and interpersonal dynamics that shape nurses’ experiences, underscoring their impact on their decisions to remain in nursing. The customized retention strategy's theme accentuates the complex landscape of nurse retention, underscoring the necessity of analyzing nurses’ profiles to understand their lifecycle and career stage needs.

The findings align with Kanter's Structural Empowerment Theory, which emphasizes access to resources, support, information, and opportunity as determinants of workplace engagement and retention (Kanter, 2008). Our synthesis suggests that mid-career nurse retention is strengthened when organizations intentionally create conditions that enhance perceived control, professional growth, and meaningful contribution.

While leadership support and professional development are common retention factors across nursing career stages, the literature on mid-career nurses places particular emphasis on career mobility, diversification of roles, and autonomy as key to sustained engagement.

The effectiveness of examined interventions in improving mid-career nurse retention remains uncertain, as many studies offered minimal reporting on outcomes or specific metrics at both individual and organizational levels, with limited discussion on unintended consequences or unanticipated results. Nonetheless, some authors conducted formal assessments, including evaluations (Allen, 2017; Torres et al., 2024), pre-and post-assessments of vacancy rates (Evans, 2017), balanced scorecards with strategy maps (Embree et al., 2015), comparisons of turnover rates between participants and non-participants (Gilroy et al., 2020), measuring the number of retained nurses (Dawood & Gamston, 2019), and reductions in turnover intentions (Guo et al., 2020).

However, while these studies provided some evidence on efficacy retention projects, it is challenging to generalize strategies to other healthcare settings. Only three articles discussed scalability, one focused on a transition program to another unit (Gilroy et al., 2020), another on professional development for nursing specialist roles (Evans, 2017), and the third on improving organizational retention through nurse career advancement programs (Torres et al., 2024). Furthermore, despite evidence highlighting the significance of rewards, recognition, and wellness activities in nurse retention (Baljoon et al., 2018; National Health Service Employers, 2024), few projects focused on these factors, with only one addressing rewards (Kuykendall et al., 2014) and another focusing on wellness programs (Guo et al., 2020).

Notably, external push and pull factors, such as caregiving responsibilities, wage progression, geographic mobility, and broader socioeconomic conditions, were not addressed in included studies. While these factors are widely recognized in nurse workforce literature (Huang et al., 2025), they remain underintegrated into mid-career–focused retention strategies. Future research should consider how organizational interventions intersect with life-stage transitions and external labor market dynamics.

A significant gap identified in this review is the lack of a clear concept and universal definition of mid-career nurses within the reviewed literature. In particular, finding literature on retaining mid-career nurses proved challenging, as many articles did not explicitly mention mid-career or experienced nurses in their titles.

The definitions of mid-career nurses across the reviewed literature varied significantly, contributing to ambiguity in the identification of this cohort. Terminology, such as experienced, mid-career, expert, and seasoned nurses, was used interchangeably, while mid-career and late-career retention strategies were often merged (Bélanger-Hardy et al., 2024). Some authors provided vague descriptions recognizing mid-career nurses as those who have been with the organization for a year or two (Knighten & Yvanovich, 2023). Others excluded participants with less than 3 years (Dawood & Gamston, 2019) or less than 6 months of experience (Gilroy et al., 2020) from their study samples of mid-career nurses.

Several authors classified mid-career nurses based on years of experience, such as more than 5 years (Hayward et al., 2016; Wardhani & Hariyati, 2023; Yarbrough et al., 2017), more than 6 months in an acute care setting (Rogers & Burke, 2021), more than 3 years working as a nurse (Ko et al., 2024; Seller-Boersma et al., 2023) or 5–15 years of experience (Shin & Lim, 2021). Some papers relied on the age of the nursing workforce to categorize mid-career nurses as belonging to the 35–50-year-old age group (Bélanger-Hardy et al., 2024) or 45 years or older with at least 10 years of experience (Kuykendall et al., 2014).

The lack of consistency in terminology emphasizes the need for a standardized and universally accepted definition of mid-career nurses in order to facilitate clearer research outcomes and targeted retention strategies. Future research should consider a dedicated scoping review to explore and consolidate existing definitions, offering a more systematic exploration of this topic.

Our review is subject to several limitations. First, although we developed a working definition of the mid-career nurse to guide our search process, we recognize that considerable variation exists in how this cohort is described across the literature. To capture the breadth of relevant evidence, we included studies that referred to nurses as experienced, seasoned, expert, or mid-career. We excluded only studies that focused exclusively on early- or late-career nurses or lacked information on job tenure. Nevertheless, our operational definition may have influenced the selection of included studies and, consequently, the interpretation of the findings.

Second, we restricted our search to papers published in English after January 2014, potentially overlooking insights from non-English sources and older publications. Third, all included studies were conducted in high-income countries, which may limit the transferability of findings to low- and middle-income country (LMIC) settings where health system contexts and workforce challenges may differ. Fourth, a comprehensive systematic search of grey literature was not performed. While selected grey literature sources were consulted, think tank and nursing association reports were not systematically searched, which may have resulted in the omission of additional relevant evidence.

Fifth, no formal quality appraisal was conducted, which is consistent with Levac et al.'s (2010) guidance. We acknowledge that not assessing the studies’ quality may influence the robustness of our findings. Lastly, due to limited data availability, we were unable to assess or compare the effectiveness of interventions, highlighting the need for further research in this area.

Included studies spanned pre-pandemic, pandemic, and early post-pandemic periods. However, no studies explicitly evaluated retention strategies designed in direct response to COVID-19 workforce pressures. Comparative evidence examining pre- and post-pandemic intervention effectiveness was notably absent, representing an important gap for future research.

## Conclusion

This scoping review provides a broad mapping of the current grey and peer-reviewed literature regarding retention strategies for mid-career experienced nurses, shedding light on existing gaps and suggesting avenues for future research. By analyzing the existing interventions and definitions, we advocate for greater clarity in mid-career nurses’ terminology. A universal definition would facilitate a deeper exploration of nurse profiles, the identification of their needs, and the development of tailored retention policies and strategies.

The review revealed a scarcity of research studies and emphasized the necessity for more experiments in the field. It highlighted the need for formal evaluations of existing projects and gathering nurses’ perspectives on the effectiveness of these initiatives.

It is imperative to shift from asking “what could work” to examining “how it works” and “whether it can be scaled” across healthcare settings, drawing on established implementation science frameworks, such as, for example, CFIR (Damschroder et al., 2022) and RE-AIM (Glasgow et al., 2019), to guide evaluation and adaptation. This shift in focus will facilitate the development and implementation of effective and scalable retention strategies for mid-career nurses, enhancing healthcare workforce stability and improving patient care outcomes.

## Supplemental Material

sj-docx-1-cjn-10.1177_08445621261463085 - Supplemental material for Exploring Retention Strategies for Mid-Career Nurses: A Scoping Review and Thematic Analysis Supplemental material, sj-docx-1-cjn-10.1177_08445621261463085 for Exploring Retention Strategies for Mid-Career Nurses: A Scoping Review and Thematic Analysis by Victoria Babysheva and Elena Neiterman in Canadian Journal of Nursing Research

## Data Availability

No new data were created or analyzed in this study. Data sharing is not applicable to this article.
